# Fate of Antibody-Drug Conjugates in Cancer Cells

**DOI:** 10.1186/s13046-017-0667-1

**Published:** 2018-02-06

**Authors:** Cécile Chalouni, Sophia Doll

**Affiliations:** 1 Department of Pathology, Genentech, San Francisco, CA USA; 2 Max Planck Institute, Munich, Germany

**Keywords:** Antibody-drug conjugates, Endocytosis, Endocytic compartments, Intracellular trafficking

## Abstract

Antibody-Drug Conjugates (ADCs) are a class of cancer therapeutics that combines antigen specificity and potent cytotoxicity in a single molecule as they are comprised of an engineered antibody linked chemically to a cytotoxic drug. Four ADCs have received approval by the Food and Drug Administration (FDA) and the European Medicine Agency (EMA) and can be prescribed for metastatic conditions while around 60 ADCs are currently enrolled in clinical trials. The efficacy of an ADC greatly relies on its intracellular trafficking and processing of its components to trigger tumor cell death. A limited number of studies have addressed these critical processes that both challenge and help foster the design of ADCs. This review highlights those mechanisms and their relevance for future development of ADCs as cancer therapeutics.

## Background

Cancer remains the 2nd leading cause of death globally (reported by the Centers for Disease Control and Prevention, http://www.cdc.gov/cancer/dcpc/data/types.htm). Metastatic disease is the most common cause of cancer-related mortality and remains a therapeutic challenge. For the past two decades, engineered monoclonal antibodies have provided a clinical approach to specifically target cancer cells, and are often combined with chemotherapy. The clinical advantage of these therapeutic antibodies comes from the fact that they target surface antigens that are expressed at equivalent or higher levels with higher or equivalent expression levels on tumor as compared to normal cells, but side effects associated with elimination of the normal cell population is clinically manageable. Anti-tumor efficacy can be achieved through downstream signaling events such as growth and proliferation inhibition initiating apoptosis [1] or by activating the patient’s immune system, resulting in complement or antibody dependent cellular cytotoxicity (ADCC) [2].

**Fig. 1:**
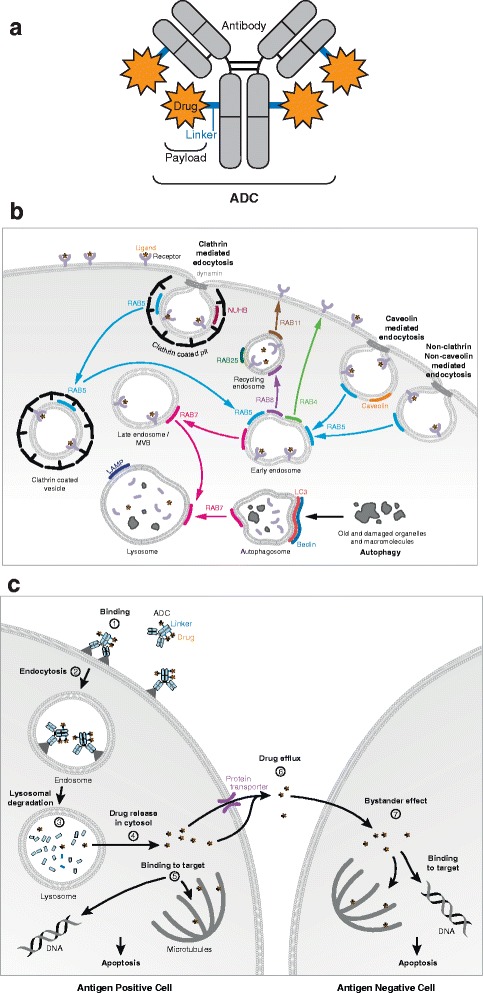
**a** - ADC structure: An ADC is composed of an antibody coupled to cytotoxic drugs by linkers. **b** - ADC trafficking and processing classic model: The ADC binds to its surface antigen (1) and the complex is internalized (2), it reaches lysosomes where its linker of the ADC is degraded leading to the release of the drug (3), the drug passes from the intracellular compartment to the cytosol (4), and binds to its target, DNA or tubulin (5) ensue apoptosis. It may also be released into the microenvironment via pumps or passive transfer through the cell membrane (6), capacity to enter a neighbor cancer cell (7) resulting in bystander effect. **c**- Endocytosis and autophagy pathways

Several therapeutic antibodies have been redeployed as the antibody component of antibody-drug conjugates (ADCs). While these antibodies primarily function as carriers of the drug to the cancer cells, some of them, like Trastuzumab in T-DM1 also act through ADCC. ADCs are composed of a humanized or chimeric antibody chemically linked to a cytotoxic drug (Fig. 1a) allowing the delivery of cytotoxic drug specifically to antigen-positive malignant cells [3]. The antibody’s specificity and the local release of cytotoxic drug are the main parameters that provide increased anti-tumor efficacy and decreased systemic toxicity. Therefore, ADCs have a wider therapeutic window compared to traditional chemotherapy [4, 5]. A chemical linker is used to attach the cytotoxic drug to the antibody, and the physicochemical properties of the linker largely determine the pharmacokinetics of an ADC [6, 7]. Linkers are usually classified as “cleavable” and “non-cleavable”. They are designed to be highly stable in the circulation to limit systemic toxicity and to be readily cleavable once the ADC reaches its intracellular destination to deliver the payload [8]. “Cleavable” linkers are designed to allow the release of the drug by hydrolysis (low pH, reduction of disulfide bonds) or by proteolysis. Linkers designed for proteolysis contain sites specifically recognized by certain enzymes, such as cysteine proteases. “Non-cleavable” linkers rely on the degradation of the antibody itself to release their cytotoxic payload. There are two main categories of cytotoxic drugs [9]: microtubule inhibitors [10] and DNA damaging drugs [11–13]. The first generation of ADCs consisting of conventional chemotherapy drugs linked to monoclonal antibodies was short lived because of insufficient potency of the drug or the instability of the ADC. A first generation ADC, gemtuzumab ozogamycin (GO/Mylotarg) targeting CD33 was approved by the FDA in 2000 to treat acute myeloid leukemia (AML). It was removed from the market due to toxicity in 2010 and reintroduced in 2017 after revising the dosage, and course of treatment (FDA news release, 2017). 2nd generation ADCs use more potent drugs (100–1000 fold). Commonly used drugs are derived from maytansinoids such as emtansine (DM1) [14], mertansine (DM4) [15] and auristatins such as mono-methyl auristatin E (MMAE), and mono-methyl auristatin F (MMAF). They induce cell death through the depolymerization of microtubules [10]. DNA damaging drugs can be also incorporated: some intercalate into the DNA (doxorubicin) blocking the activity of the topoisomerase 2, others can cleave (calicheamicin) [16], alkylate DNA (DGN462) [17], or inhibit enzymes associated with DNA damage (topoisomerase inhibitor SN38) [18]. The stochastic coupling of the drugs in 2nd generation ADCs results in an approximate drug: antibody ratio (DAR) around 4:1. Three 2nd generation ADC molecules brentuxumab vedotin, trastuzumab (described below) were given FDA approvals respectively in 2011 and 2013. Inotuzumab ozogamycin, that targets CD22, received FDA approval in 2017 to treat relapsed or refractory B cell precursor acute lymphoblastic leukemia.

The third generation of ADCs is designed to have a wider therapeutic window. The main difference compared to 2nd generation ADCs, is the site-specific conjugation of the drug that allows a better control of the DAR and results in better in vivo efficacy and safety [19]. In addition, these new ADCs can contain improved warheads and incorporate new antibody structures such as biparatopic antibodies [20]. Interestingly, two-thirds of the ADCs currently enrolled in clinical trials have maytansinoid or auristatin cytotoxic warheads [20].

Antigen binding, the anti-tumor potency of cytotoxic drugs and the favorable pharmacokinetic profile of the antibody are critical for the function of the ADC. In addition the efficacy of an ADC also relies on its internalization and processing, the release of the drug from the intracellular compartment to the cytosol and binding to its intracellular target to trigger cell death (Fig. 1b).

The ADC internalization route is usually described as following the endocytosis pathway relevant to antibodies and receptors bound to their ligands [21].

The general concept of the trafficking and processing of ADCs in the endocytic pathway (Fig. 1b) is that once an ADC binds to its surface tumor antigen, it is internalized into endosomes that subsequently mature and fuse with lysosomes [22, 23]. In the lysosomes, the drug is released via cleavage of the linker by specific proteases such as cathepsin B or by the degradation of the ADC. The freed drug traverses the lysosomal membrane and reaches the cytosol and will bind to its target, either the microtubules [4] or DNA and trigger cell death. More recently, in vitro studies have shown that the free drug in the cytosol can cross the plasma membrane to access the extracellular milieu and kill neighbor cells by a process called the bystander effect [24–26]. This additional outcome is increasingly taken into account in the design of ADCs since it implies that only a subset of antigen expressing cells are needed in order to target the drug to a larger tumor population.

More specifically, receptor mediated endocytosis has been extensively studied in both normal and cancer cell types [27–29] and can occur through a clathrin-mediated, caveolin-mediated or clathrin and caveolin independent mechanisms (see Fig. 1c) [30]. The clathrin dependant pathway is defined as the route ftaken up by most antigen-antibof = dy complexes. Trough this pathway, the ligand-receptor complexes cluster into pits where clathrin self assemble into lattices that drive membrane invagination. Dynamin, a GTPase whose major function is to control fission of endocytic vesicles [31], self-assembles at the neck of the pit and triggers the fission of the vesicle from the plasma membrane. The newly formed clathrin coated vesicles mature, losing the clathrin coat, and fuse together to form early endosomes (pH: 5.9–6). From the early endosomes, the cargo can be directly recycled back to the surface through a fast recycling route relying on the activity of the small GTPase Rab4, or via recycling endosomes and through the combined activities of Rab8 and Rab11 [32]. Alternatively, early endosomes can mature into more acidic late endosomes that ultimately fuse with lysosomes through Rab7 [33]. Lysosomes function at the crossroads of endocytosis, autophagy [34] (Fig. 1c) and phagocytosis. They are characterized by an acidic pH (4–5) and high content of protein digestive enzymes such as cathepsins and collagenases among many others (nucleases, lipid digesting enzymes…). Their low pH is maintained through ATP-ases that pump protons from the cytosol to the lysosomal lumen [35].

Along with material uptake, endocytosis regulates signal transduction as well as cell adhesion and migration. While genetic alterations of membrane trafficking genes are rare, alterations in expression levels are frequently reported. These modifications can result in epithelial-mesenchymal transformation (EMT), metastasis and cancer stem cell emergence. Clathrin, caveolin, Rabs (Rab4, Rab5A, Rab1A, Rab2A) as well as their effectors (Rab25, Vav1, Rab coupling protein,cdc42, VSP39), can contribute to the processes of cancer cell survival and metastasis, and cancer stem cell emergence [36–38] (Table 1). In addition, lysosomes have been found to contribute to hallmarks of cancer such as proliferation, invasiveness, angiogenesis, and metastasis [39].

**Table 1:** Roles of molecules involved in endocytosis in normal and cancer cells. Figure adapted from L. Lanzeti et al

Molecule	Role in normal cells	Role in Cancer cells	Alterations	Tumor type
Clathrin	Endocytic vesicle formation at PM Cell division Transport of extracellular materials	Tumor suppressor	M, T	Lymphoma Kidney
Dynamin 2	Fission of endocytic vesicles	Migration Invasiveness	O	Pancreas
Rab5A	Vesicle formation Vesicle movement Vesicle maturation Trafficking of metalloproteases	Invasiveness Extension of invadosomes Conversion of in situ carcinomas to invasive ones	O	Breast
Rab4	Recycling to PM	Invasiveness	O	Breast
Rab11 and effectors	Recycling of Tight junctions, EGFR and integrins to PM	Invasiveness Prediction of metastasis	O	Breast
Rab 1A	Sensing of amino acids	Proliferation Cellular transformation	O	Colon
Rab7	Formation of autolysosomes Fusion of phagosomes with lysosomes Trafficking to lysosomes	Tumor suppressor	na	
Numb	Maintenance of stem cell compartments Formation of endocytic vesicles Recycling to PM	EMT Emergence of CSC	U	Breast Lung
Rab2A	Fusion of late endosomes with lysosomes Recycling of metallo-proteases to PM Controls ER-Golgi transport	Invasiveness EMT Expansion of CSC compartment	O	Breast
Caveolin	Coat protein Signaling Transport of membrane components and growth receptors	Loss of caveolae results in cell proliferation	U, M, O	Multiple cancers
LAMP1	Protein of lysosome membrane	Expression levels correlated with invasiveness	O	Melanoma Colon
CD71	Receptor to transferrin	Loss of CD71 correlates with expansion of CSC and invasiveness	O U	Colon Pancreas Stomach Pancreas

*PM* Plasma membrane, *M* Mutated, *T* Translocation *T O* Overexpressed, *A* Amplified, *U* Under-expressed, *EMT* Epithelial- mesenchymal transition, *CSC* Cancer stem cells, *ER* Endoplasmic reticulum, *NA* Not available

While the design of an ADC may rely on features specific to the trafficking of the parent antibody [21], this strategy can be challenging as the addition of a drug can result in subtle differences in these antibody properties such as preventing or inducing the internalization of a construct as seen for anti-CD19 antibody [40–43].

Noteworthy, hardly any data is published about trafficking and processing of 1st generation ADCs and FDA approved 2nd generation ADC, inotuzumab ozogamicin (CMC544/Besponsa^TR^). It is assumed they undergo receptor mediated endocytosis similar to the constituent antibody and summarized as the classic model of ADC trafficking and processing (Fig. 1c). However, trafficking, processing, and bystander effect of 2nd and 3rd generation ADCs have been studied. In the following sections, we summarize those results that shed light on the complex and unique interactions between ADCs and tumor cells and how these observations contribute to optimize ADCs design for increased efficacy [21, 29, 44].

### 1-trafficking and processing of second generation ADCs

#### A- anti-CD30 mAb based ADC: The example of Brentuximab vedotin (Adcetris™)

Brentuximab vedotin was approved for treatment of relapsed or refractory anaplastic large cell lymphoma in 2011. Brentuximab vedotin; SGN-35) is a chimeric monoclonal anti-CD30 antibody cAC10 bound to the auristatin agent MMAE via an enzymatic cleavable valine-citrulline (vc) – paminobenzyl alcohol (PAB) linker, with a DAR of 4:1 [45]. The vc linker is specifically designed to be cleaved by the lysosomal enzyme, cathepsin B. CD30 is a transmembrane protein of the TNF receptor 3 superfamily restrictively expressed on activated B and T cells at low levels in normal tissues and over-expressed on their malignant counterparts in Hodgkin lymphoma (HL) and anaplastic large cell lymphoma [46].

Trafficking and processing of cAC10 was initially studied with the antibody bound to MMAE or MMAF via a vc linker [47]. These constructs as well as the relevant naked antibody have comparable surface labeling of CD30+ cell lines and similar internalization rates. As measured by flow cytometry, 60% of the initial level of surface bound cAC10 antibody remained after 20 h. Intracellular levels rose to a plateau of around 15% of the total membrane signal by 5 h, and were maintained until the last time point at 20 h. When cAC10 was cross-linked with anti-human IgGs, it resulted in a 3-fold increase of intracellular antibodies. This shows that higher level of clustering of the ADC-antigen complexes results in greater internalization. cAC10 and corresponding ADCs have been shown to be internalized via clathrin coated pits as blocking this pathway decreased internalization. When visualized by microscopy, the three different cAC10 antibody constructs reached acidic compartments labeled with lysotracker after 3 h of incubation. At 20 h, anti-LAMP1 staining allowed detection of the three constructs within lysosomal compartments. The presence of the ADCs within the lysosomes was confirmed by isolating organelles using gradient fractions. Western blots detecting the drug and the heavy chain of the ADCs showed that the drug release was prevented when cells were treated with cysteine proteases inhibitors consistent with a critical role of proteases, such as cathepsin B, in cleaving the vc linker.

While the trafficking and kinetics of the MMAF- and MMAE-conjugated ADCs appear similar, the cytotoxicity of MMAF-conjugated ADC is 2 to 3 fold higher compared to MMAE-conjugated ADC [47]. This suggests subtle differences in the intracellular processing of these ADCs and their components. Free MMAE is 200 times more cytotoxic than MMAF when added directly to the cell cultures as uncharged MMAE traverses plasma membranes more efficiently than the charged MMAF. These data suggest that the intracellular route favors the potency of the MMAF conjugated ADC while MMAE conjugated ADC may have a better bystander effect.

Okeley et al. [48] measured the drug release and studied the bystander effects of Brentuximab prepared with ^14^C–MMAE (^14^C- SGN35) using a combination of radiometric and liquid chromatography and mass spectrometry based assays. High intracellular MMAE concentrations (>400 nmol/L) were detected within 24 h of continuous treatment with ^14^C- SGN35 of CD30 positive cell lines such as L540cy HL and Karpas 299 anaplastic large cell lymphoma cells. Cytosolic MMAE concentrations were found to be sustained, while it also accumulated in the culture medium. This suggests a constant internalization and processing of the ADCs while the free drug traverses the plasma membrane into the extracellular milieu. 2.5 ADCs per receptor were found to be internalized and catabolized by Karpas 299, and 3.4 ADCs per receptor by L540cy cells after 72 h of incubation suggesting recycling or the newly synthesized CD30 reaching the surface. In contrast to Ly540 cells, Karpas 299 cells were found to express multidrug resistance proteins (MDRs) as shown by efflux of rhodamine dye. Therefore, while MDR efflux pumps contribute to drug efflux in Karpas cells, extracellular drug release in Ly540cells may be primarily due to diffusion. The bystander effect of extracellular MMAE was observed in cocultures of Karpas 299 or Ly540 cells in presence of SGN35 with CD30 - negative Burkitt lymphoma cells (Ramos). This effect is advantageous for the treatment of HL because of the heterogeneity of its cancer cells subsets: only a small fraction is CD30 - positive [49, 50].

#### B- anti-HER2 based ADC- Trastuzumab emtansine (Kadcyla™/T-DM1) trafficking (Fig. 2)

T-DM1 received approval by the FDA in early 2013 to treat recurrent and refractory HER2-positive metastatic or locally advanced breast cancer previously treated with trastuzumab and a taxane [51, 52]. T-DM1 is comprised of a humanized monoclonal anti-HER2 antibody (trastuzumab, Herceptin™) conjugated to DM1, via an N-succinimidyl 4(N-maleimidomethyl) cyclohexane-1-carboxylate (SMCC) linker with a DAR of 3.5:1. Similar to its parent antibody, T-DM1 targets its epitope located in the juxtamembrane extracellular domain of HER2, a receptor tyrosine kinase amplified in a subset of breast, ovarian and gastric cancers [53, 54]. Pharmacokinetic analyses of trastuzumab based ADCs with different linkers in nude mice led to the choice of the non-cleavable SMCC linker that is highly stable in the circulation [55]. T-DM1 combines the ADCC signaling properties of trastuzumab with the cytotoxicity due to the drug.

**Fig. 2:**
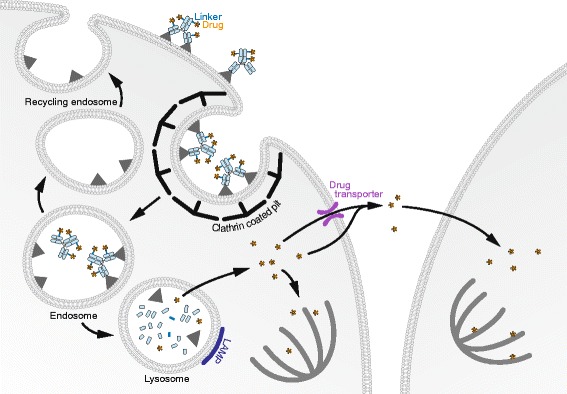
Mode of action of brentuximab-vedotin. In Hodgkin lymphoma CD30 positive cells, brentuximab-vedotin is internalized via clathrin coated pits and reaches endosomes. CD30 can recycle or be newly synthesized and re-expressed at the cell surface. The anti-CD30 ADC complex reaches lysosomes where the linker is cleaved by proteases. MMAE molecules traverse the lysosomal membrane and access the cytosol. MMAE can bind the microtubules, triggering depolymerisation leading to cell death. MMAE can traverse the plasma membrane of certain cell lines and exert a toxic effect on neighbor cells (bystander effect)

While the intracellular trafficking of T-DM1 is not published yet, trastuzumab [56] was shown by fluorescence and electron microscopy to occasionally be present in clathrin coated pits and to traffic to transferrin-positive recycling endosomes in the breast cancer cell line SKBR3. The recycling of trastuzumab bound to HER2 via the endosomes to the membrane is rapid: 50% within 5 min, and 85% within 30 min as measured by immunofluorescence. In house data generated with T-DM1 added to SKBR3 cells show that it reaches transferrin positive endosomes and confirms that this environment allows the release the drug from the antibody [2].

Diessner et al. [57] identified that T-DM1 could kill highly aggressive, poorly immunogenic breast cancer stem cells (CSC) [58] purified from a MCF7 cell line. Remarkably, this subset of cells was found to utilize autophagy to internalize trastuzumab conjugated to the fluorescent dye phrodo, in place of DM-1. Both the down regulation of the autophagy mediators Beclin and LC3, and the treatment with the autophagy inhibitor artesunate blocked T-DM1 internalization in these CSC. T-DM1 trafficking would differ in CSC compared to differentiated cancer cells and autophagy could account for the increased efficacy of T-DM1 [57].

The extracellular release of DM1 [55] was studied by using [3H] trastuzumab-MCC-DM1 internalized by BT474 EEI cells derived from BT474 breast cancer xenograft. This study showed that the moiety lysine-N^Ε^ -MCC-DM1 is the main released catabolite. The active form, lysine-MCC-DM1, does not traverse the plasma membrane of neighbor cells, hence, the bystander effect of T-DM1 may be limited [24, 59]. Barok et al. [60] propose that cytosolic concentration of lysine-MCC-DM1 released by T-DM1 determines its efficacy. Low levels seem to have no effect, while a gradual increase of lys-MCC-DM1 concentration triggers disruption of intracellular trafficking by inhibiting transport along microtubules, supported by observations in preclinical breast cancer models with low proliferation rates [61]. In xenografts, T-DM1 triggers the disruption of intracellular trafficking followed by mitotic catastrophe and cell cycle arrest in the G2-M phase, leading to apoptosis [55, 62].

A noticeable benefit of T-DM1 is to overcome certain treatment resistances. It was found to be efficacious in trastuzumab-insensitive models such as HER2 overexpressing breast cancer cell lines [55] and in HER2-expressing lung, ovarian, and gastric carcinoma cell lines [14]. The increased efficacy in these cell lines is likely due to the added cytotoxicity of DM1. Furthermore, Lewis Phillips et al. [63] found a synergistic benefit of combining pertuzumab (Perjeta™) with T-DM1 in overcoming trastuzumab resistance. Pertuzumab is an anti-HER2 antibody that prevents binding of HER2 to other HER family members. Increased sensitivity to T-DM1 was induced by perjeta which blocks binding of HER3 ligand to HER2 in a subset of cancer cell lines. This resulted in the inhibition of cell proliferation and triggering of apoptosis in vitro *and* synergistic anti-tumor activities in vivo [63]. The approach of combining pertuzumab with T-DM1 anticipated the development of a biparatopic bispecific antibody by Li et al. [64]. In addition, the finding that the catabolites of linkers-maytansinoids can be substrates for MDR1, while not having a bystander effect, resulted in generating new ADCs with different linker - drug combinations [65].

### 2-trafficking and processing of third generation ADCs

#### A - A biparatopic anti-HER2 based ADC

Crosslinking antibodies can result in increased internalization, inhibition of recycling and increased lysosomal degradation [66, 67]. All these features are advantageous for ADCs. Also, Li et al. [64] developed a bivalent anti-HER2 biparatopic antibody, encompassing four target-binding sites. Two sites on each arm can recognize different HER2 epitopes: one HER2 epitope is detected by the trastuzumab component of the molecule and the other HER2 epitope is detected by a component contributed by the anti-HER2 monoclonal antibody 39S. This biparatopic antibody was found to be capable to induce the signaling effects issued from the two original antibodies i.e. to block both ligand-independent and ligand-dependent receptor activation that drive cell proliferation of cancer cells. The biparatopic antibody was used as an ADC by conjugating it with a variant of the tubulysin peptide, which blocks mitosis and triggers cell death by depolymerizing microtubules. Increased clustering of HER2 with the biparatopic antibody compared to bivalent antibody was characterized by size exclusion chromatography combined with multi-angle light scattering analysis (HPLC SECMALS) [64]. Internalization of the biparatopic antibody along with various antibodies including trastuzumab was assessed by flow cytometry and imaging, using BT474 cells. The biparatopic ADC was found to be internalized much faster and in greater amounts than trastuzumab, pertuzumab and 39S. By immunofluorescence, the biparatopic antibody-receptor complexes colocalized with the lysosomal marker LAMP while little colocalization was found following trastuzumab-HER2 complexes internalization as in previous studies. In addition, the biparatopic antibody induced the degradation of HER2 as demonstrated by the absence of HER2 in the lysosomal fraction of cells treated with the biparatopic antibody in contrast to cell lysates treated with other antibodies. This observation suggests that the biparatopic antibody is more efficiently degraded than trastuzumab and hence may release more drug into the cytosol [64]. In vitro cytotoxicity was evaluated in a panel of human cancer cells with different levels of HER2 expression. Overall, the cytotoxicity correlated with the level of HER2 expression while the biparatopic ADC was found to be 10 times more potent than T-DM1 in HER2 overexpressing cells. In addition the biparatopic antibody could kill cell lines, either overexpressing HER2, or having low HER2 levels, that are resistant to T-DM1. In vivo data showed that the biparatopic ADC is more potent than T-DM1 in tumor models representing T-DM1 eligible and ineligible patients, or with acquired T-DM1 resistance [64]. Notably, 16 primary tumor models derived from HER2-negative breast cancer patients, including tumors with heterogeneity in expression of estrogen receptors and progesterone receptors and histological subclass, showed regression upon treatment with the biparatopic ADC. These results support the potential use of the biparatopic ADC in a patient population that is currently ineligible for HER2 targeted therapies. These outcomes could be partially explained by the bystander effect of the biparatopic ADC. In vitro*,* the biparatopic ADC could kill both HER2 expressing cells and negative cells in co-cultures in contrast to T-DM1 [64], that could probably be explained its different linker-drug composition.

#### B - anti-CD33 based ADCs: Gemtuzumab-Ozogamycin (Mylotarg™, GO), SGN-33A (Vadastuximab-talirine) and IMGN779

The first commercialized anti-CD33 based ADC, gemtuzumab-ozogamycin (GO) [16] brought some insights into the mechanism of action, but toxicity leading to increased deaths led to its removal from the market [68] in 2010. The limited efficacy of GO has been linked to the heterogeneity of the DAR and the cellular efflux of its drug, calicheamicin by MDRs. MDRs are commonly expressed in AML, and their presence predicts treatment failure [69]. However, GO was reintroduced into the market in 2017 after revising the dosage, and course of treatment (FDA news release, 2017). SGN-33A and IMGN779 are third generation ADCs that target CD33. SGN-33A consists of a humanized anti-CD33 mAb bound via a protease cleavable linker, maleimidocaproyl valine-alanine dipeptide linker to pyrolobenzodiazepine dimers (PBD) with a DAR of 2. PBD blocks cell division and induces cell death by binding and crosslinking specific sites in the DNA minor grove. SGN-33A is currently enrolled in phase III clinical trials for the treatment of Acute Myeloid Leukemia (AML) where CD33 (sialic acid-binding sialo-adhesin receptor 3) is expressed on malignant cells in the vast majority of patients.

Sutherland et al. [17] have studied SGN-33A efficacy and trafficking. SGN-33A brings significant improvement compared to GO as the linker technology provides improved conjugation of the drug allowing for increased intracellular delivery of the drug. SGN-33A is highly active (with an inhibitory concentration 2.5 times lower than that of GO) against AML cell lines and primary AML cells in vitro, and in xenotransplantation studies. By fluorescence microscopy, SGN-33A was found to internalize, and reach lysosomes after 4 h of incubation with HNT34 cells [17]. The use of PBD as a drug warhead resulted in significantly improved anti-tumor activity in the cell lines expressing MDR and previously resistant to GO. Additionally, SGN-33A was found to have significant cytotoxic effects on samples with any cytogenetic risk (unfavorable, intermediate or favorable) suggesting that this ADC may have a broad impact in treating AML [17].

More recently a new 3rd generation anti-CD33 ADC, IMGN779, was engineered with DGN462, a novel DNA alkylating agent consisting of an indolino-benzodiazepine dimer, bound via a cleavable disulfide linker. The drug was found to better balance efficacy with tolerability while the linker was designed to enhance bystander effect without increasing systemic toxicity. This ADC is enrolled in phase 1 clinical trials.

## Conclusion

**Fig. 3:**
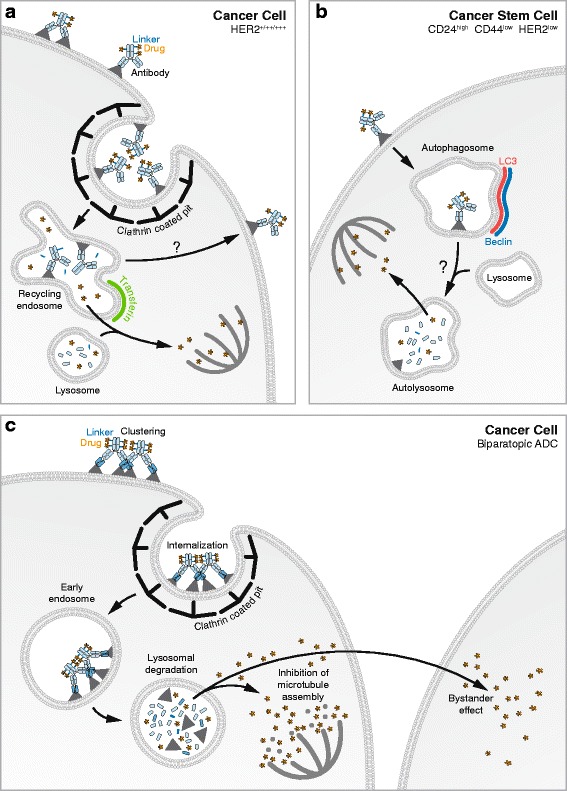
Mode of action of anti-HER2 based ADCs, T-DM1 and the Biparatopic anti-HER2 ADC. **a **- In HER2 positive cancer cells, T-DM1 is internalized via clathrin coated pits and reaches endosomes where the antibody is degraded leading to the release of DM1. A small fraction of T-DM1 reaches the lysosomes where it can also be degraded. DM1 traverse the lysosomal/endosomal membrane and accesses the cytosol. DM1 can then bind the microtubules, triggering depolymerisation leading to cell death. **b** - In cancer stem cells, T-DM1 is endocytosed via autophagy. It reaches autophagosomes and later autolysosomes where it is degraded leading to the release of DM1. DM1 accesses the cytosol and inhibits microtubules polymerization leading to cell death. **c** - The biparatopic anti-HER2 ADC has more efficient trafficking to the lysosomes compared to the monoclonal T-DM1. Its drug tubulysin is liberated in the lysosomes and accesses the cytosol where it can induce the depolymerization of microtubules. In addition, it can exert a bystander effect

The efficacy of an ADC relies on numerous factors from the characteristics of the ADC itself to the biological features of the tumor cells (Table 1). Summarized (Fig. 3), these are: the binding affinity of the antibody, the internalization of the ADC, the intracellular release of its drug or toxic moiety, the transport of the drug from the compartment to the cytosol, the drug cytosolic concentration, its capacity to trigger cell death and a bystander effect. In addition to the techniques described in the review to assess the intracellular fate of ADCs, new strategies to track ADC components in cells and in the extracellular milieu and are continuing to be developed. For example, BC Lee et al. developed a novel Fluorescence Resonance Energy Transfer (FRET) approach that was used to track antibody-antibiotic conjugates (AAC) inside macrophages [70]. The FRET labeled AAC allowed the tracking of the intracellular linker cleavage of the antibiotic vancomycin, from an anti-*Staphylococcus aureus* antibody. This technology is currently being applied to ADCs (manuscript in preparation) to further understand the trafficking and processing of the ADC and its constituents.

As it is usually envisioned, the trafficking of ADCs seems to typically result in the liberation of the drug within the clathrin mediated endocytic pathway as observed for brentuxumab vedotin, T-DM1, as well as the anti-CD19 based ADC hBU12-vc-MMAE [42]. Commonly, the place of degradation of an ADC has been found to be in lysosomes as showed for SGN-A33 [17], and the anti-CD79b based ADC, hBU12-vc-MMAE, polatuzumab vedotin [71]. However, in certain cases, endosomes may provide an environment that allows the release of the drug from its antibody as suggested by studies of T-DM1 internalization by breast cancer cells. The question of targeting a specific intracellular compartment remains an area of investigation for improved efficacy. Interestingly, in the case of anti-HER2 based ADCs, the biparatopic anti-HER2 ADC [64] has proved to be more efficacious in vivo than T-DM1 while mostly being found to reach lysosomes and not endosomes. Additionally, Diessner et al. [57] found that T-DM1 could be found in autophagic compartments in breast cancer stem cells. It is noteworthy that CSCs rely on autophagy for their survival [72, 73]. They have been described to be associated to cancer initiation, propagation, spreading, and recurrence and are often resistant to cancer therapies. Hence targeting CSCs for cancer treatment may be valuable [74]. An ADC developed to target cancer stem cells is Rovalpituzumab Tesirine (Rova-T) [75]. It aims to treat recurrent small cell lung cancers (SCLC) and possibly the large cell neuroendocrine carcinomas (LCNEC). It is based on an anti-DLL3 antibody, and was found to reach stomatin-like protein-1 (SLP-1) positive compartments of DLL3 transfected cells. SLP-1 [76] is a marker for late endosomes as well as lysosomes, not clearly answering the question of the route taken by the ADC. It would be of interest to study more thoroughly the trafficking and processing of ADCs in CSCs and differentiated cancer cell subsets in vitro and in vivo, as lysosomes are both accessible via receptor mediated endocytosis and the autophagy pathway. This may further contribute to the understanding of their mechanisms of action, and tumor cell resistances.

The transport of the released drug from an intracellular compartment to the cytosol is an important factor for ADC efficacy to reach a potent cytotoxic concentration in the cytosol. Intracellular compartments have different characteristics of permeability towards drugs. For example, Hamblett et al. [77] have identified that SLC46A3, a lysosomal transporter, is required for metabolites derived from the degradation of MMAE based non-cleavable ADCs to reach the cytosol. In contrast the efficacy of MMAF based non-cleavable ADCs are not affected by silencing SLC46A3. This shows the impact of the ADC trafficking and choice of the drug for its efficacy. Another study [78] found that lysosomal V-ATPase (H + -ATPase) activity is essential to maintain the acidity of the lysosomes and allow the metabolism of T-DM1. However, the change of a non-cleavable linker and the drug DM1 for a protease cleavable linker and MMAE alleviated the resistance.

It has been commonly observed that cancer cells can develop resistance towards specific ADCs by increasing MDR expression [79, 80]. Modifying the linker and payloads of ADCs [65] can overcome MDR resistance mechanisms. Yu et al. [81] identified that changing MMAE for anthracycline overcomes the resistance in non- Hodgkin lymphomas to MMAE based ADCs, polatuzumab-vedotin (anti-CD79 based ADC) and pinatuzumab-vedotin (anti-CD22 based ADC).

The bystander effect is a desirable trait. ADCs design is evolving towards this application using new linkers and wardheads, that increase the bystander potency [25].

Finally, since molecules involved in endocytosis and autophagy can be deregulated, they can be considered as potential therapeutic targets [82–84]. Lysosomes have been found to be larger, more fragile and more sensitive towards permeabilization in cancer cells. LAMP1 can be overexpressed at the surface of cancer cells while its location is usually to the endosome-lysosomal membrane in normal cells with sometimes a low amount (1–2%) at the membrane [85]. This atypical expression is correlated with metastatic potential in melanoma cells [86] and metastatic colon cancer cells [87]. Currently, an anti-LAMP1 ADC (SAR428926-SPDB-DM4) is enrolled in phase II clinical trials for solid tumors. Another target of the endocytic pathway for an ADC is the transferrin receptor, CD71. While it is expressed at low levels by normal cells and is internalized in recycling endosomes, it is overexpressed at the membrane of certain tumors [88, 89]. AbGn107, an ADC based on an anti-CD71 (transferrin receptor 1) antibody is currently in phase I clinical trials to treat colorectal, pancreatic and stomach cancers. Little information is available regarding the two above cited ADCs. These two examples show how endocytic proteins mislocated in cancer cells can become targets of ADCs. Overall, ADCs trafficking and processing have been described in cancer cells (Table 2). A question is how this trafficking and processing would affect normal cells. Further research in this area would allow to better understand ADCs’ physiological effects.

**Table 2:** Trafficking, drug intracellular targets and bystander effects of ADCs that have received FDA approval or are enrolled in Phases II or III clinical trial

Name	Antigen	Receptor mediated endocytosis				Autophagy	Bystander effect	Target (drug)	Indication (stage)
		CCV	RE	EE	LE-Lys				
SGN35 Brentuxumab Vedotin	CD30	+	na	na	+	na	+	Tubulin (MMAE)	ALCL and HK (FDA approved 2011)
T-DM1 Trastuzumab Emtensine	HER2	+	+	na	–	+	–	Tubulin (DM1)	HER2+ metastatic breast cancer (FDA approved 2013)
SGN33	CD33	na	na	na	+	na	na	DNA (PBD)	
IMGN779	CD33	na	na	na	na	na	+	DNA (IDB))	
ROVA-T	DLL3	na	na	na	+	na	na	DNA (PBD)	
CDX-011* Glemtumumab Vedotin	GPNMB	na	na	–	–	+	na	Tubulin (MMAE)	Melanoma, osteocarcinoma (Phase II)
RG7596 Polatuzumab Vedotin	CD79b	na	na	na	+	na	na	Tubulin (MMAE)	NHL (Phase II)
SGN-CD19A* Denintuzumab mafodotin	CD19	+	na	na	+	na	na	Tubulin (MMAF)	NHL (Phase II)
PSMA ADC*	PSMA	+	+	na	na	na	na	Tubulin (MMAE)	Prostate cancer (Phase II)
IMGN853 Mirvetuximab soravtansine	FOLR1	na	na	+	na	na	+	Tubulin (DM4)	Ovarian cancer (Phase III)
SAR3419 Coltuximab ravtansine	CD19	na	na	+	na	na	–	Tubulin (DM4)	Diffuse Large B cell lymphoma (Phase II)
IMGN529 Naratuximab emtansine	CD37	na	na	na	na	na	na	Tubulin (DM1)	NHL (Phase II)
BT-062 Indatuximab ravtansine	CD138	na	na	na	na	na	+	Tubulin (DM4)	Multiple myeloma (Phase II)
Bay 94–9343 Anetumab ravtansine	Mesothelin	na	na	na	+	na	+	Tubulin (DM4)	Mesothelin expressing tumors (phase II)
SAR408701	CEACAM5	na	na	na	na	na	na	Tubulin (DM4)	Solid tumors (Phase II)
SAR428926	LAMP1	na	na	na	na	na	na	Tubulin (DM4)	Solid tumors (Phase II)

Legend for abbreviations: *CCV* Clathrin coated vesicles; *RE* Recycling endosomes; *EE* Early endosomes; *LE* Late endosomes; *LE-Lys* Late endosomes and lysosomes; *MMAE* Mono-methyl auristatin E; *MMAF* mono-methyl auristatin F; *DM1* Emtansine; *DM4* Mertansine

In conclusion, the complex heterogeneity of cancer cells and their increased mutation rates remain a challenge for the efficacy of ADCs [37]. While the expectation of delivering a “magic bullet” to tumor cells in the form of an ADC may not be fully realized yet, new generations of ADCs are developed based on advances in antigen identification, linkers technology and warheads [18]. Like with other targeted therapies, combination of different modalities may prove superior. The evaluation of combinations of ADCs with other antibodies, small molecule inhibitors and even immune checkpoint inhibitors are in very early stages [90]. Such approaches are destined to provide exciting clinical data in the future with the expectation to not only prolong survival but also improve quality of life of cancer patients.

## Abbreviations

AAC: Antibody-antibiotic conjugate
ADC: Antibody-Drug Conjugate
AML: Acute Myeloid Leukemia
ATP: Adenosine tri-phosphate
CSC: Cancer stem cell;
DAR: Drug antibody ratio
DM1: Emtansine
DM4: Mertansine
FRET: Fluorescence energy transfer
GO: Gemtuzumab-ozogamycin
HL: Hodgkin Lymphoma
HLPC SECMALS: Multi-angle light scattering analysis
LCNEC: Large cell neuroendocrine carcinomas
MDR: Multidrug resistance protein
MMAE: Mono-methyl auristatin E
MMAF: Mono-methyl auristatin F
PAB: Paminobenzyl alcohol
PBD: Pyrolobenzodiazepine
SCLC: Small cell lung cancer
SLC46A3: Solute carrier family 46 member 3
SLP-1: Stomatin-like protein 1
SMCC: N-succinimidyl 4(N-maleimidomethyl) cyclohexane-1-carboxylate
Vc: Valine-citruline
